# Chronic paternal alcohol exposures induce dose-dependent changes in offspring craniofacial shape and symmetry

**DOI:** 10.3389/fcell.2024.1415653

**Published:** 2024-07-01

**Authors:** Samantha L. Higgins, Sanat S. Bhadsavle, Matthew N. Gaytan, Kara N. Thomas, Michael C. Golding

**Affiliations:** Department of Veterinary Physiology and Pharmacology, School of Veterinary Medicine and Biomedical Sciences, Texas A&M University, College Station, TX, United States

**Keywords:** craniofacial dysgenesis, geometric morphometric, epigenetics, toxicology, dose-response, paternal epigenetic inheritance

## Abstract

Although dose-response analyses are a fundamental tool in developmental toxicology, few studies have examined the impacts of toxicant dose on the non-genetic paternal inheritance of offspring disease and dysgenesis. In this study, we used geometric morphometric analyses to examine the impacts of different levels of preconception paternal alcohol exposure on offspring craniofacial shape and symmetry in a mouse model. Procrustes ANOVA followed by canonical variant analysis of geometric facial relationships revealed that Low-, Medium-, and High-dose treatments each induced distinct changes in craniofacial shape and symmetry. Our analyses identified a dose threshold between 1.543 and 2.321 g/kg/day. Below this threshold, preconception paternal alcohol exposure induced changes in facial shape, including a right shift in facial features. In contrast, above this threshold, paternal exposures caused shifts in both shape and center, disrupting facial symmetry. Consistent with previous clinical studies, changes in craniofacial shape predominantly mapped to regions in the lower portion of the face, including the mandible (lower jaw) and maxilla (upper jaw). Notably, high-dose exposures also impacted the positioning of the right eye. Our studies reveal that paternal alcohol use may be an unrecognized factor contributing to the incidence and severity of alcohol-related craniofacial defects, complicating diagnostics of fetal alcohol spectrum disorders.

## Introduction

Fetal alcohol spectrum disorders (FASDs) are a collection of developmental anomalies and disabilities attributed to the maternal consumption of alcohol during pregnancy (Hoyme et al., 2016). These deficits range from problems with learning and behavior to the severe facial dysmorphia, central nervous system patterning defects, and growth restriction associated with fetal alcohol syndrome (FAS) (Suttie et al., 2013; Hoyme et al., 2016). Despite advances in our understanding of this condition over the past 50 years, the factors contributing to the wide variation in FASD penetrance and severity remain poorly defined.

Although exclusively attributed to maternal alcohol use, emerging clinical and preclinical studies demonstrate that paternal drinking can also cause alcohol-related behavioral, cognitive, and structural defects (Terracina et al., 2022; Rice et al., 2024). For example, recent studies in humans demonstrate that paternal alcohol use is associated with higher instances of offspring congenital heart defects and a higher mortality risk during the transition from birth into early adulthood (Zhang et al., 2020; Lee et al., 2024). Further, preconception paternal alcohol use is associated with higher odds of microcephaly, while conversely, maternal alcohol consumption before pregnancy is not (Zuccolo et al., 2016). Despite these observations, all alcohol messaging targets women (Lyall et al., 2021; Chang, 2023). Therefore, researchers, clinicians, and policymakers need to reevaluate the exclusive emphasis on maternal alcohol exposures and extend their analyses to consider paternal effects (Sharp et al., 2018).

In clinical studies and mouse models, the severity of alcohol-related changes in craniofacial shape and symmetry correlate with the dose of maternal alcohol exposure and correspond with the severity of patient diagnosis (Suttie et al., 2013; Suttie et al., 2017; Petrelli et al., 2018; Suttie et al., 2018). Whether paternal alcohol use also contributes to the emergence and severity of FAS-related craniofacial phenotypes in humans is unknown. However, using a mouse model, we recently described the impacts of chronic preconception paternal alcohol exposures on craniofacial growth and patterning, including increased incidences of microcephaly and alterations in patterns of facial symmetry, including micrognathia and retrognathia (deficient growth of the lower jaw and mandible) (Thomas et al., 2023). Based on these previous studies, we hypothesize that graded male alcohol use contributes to the incidence and severity of alcohol-related craniofacial defects. Indeed, we posit that paternal alcohol use may be an unrecognized factor complicating emerging efforts employing imaging-based diagnostics of FASDs (Roomaney et al., 2022).

Dose-response assessments represent a critical feature of developmental toxicology, establishing the graded relationships between toxicants and the associated adverse health outcomes under study. Although well-established in studies examining maternal exposures, few studies have examined dose-response relationships between paternal exposures, environmentally-induced alterations in developmental programming, and the impacts on offspring phenotypes. Early studies in rats examining the impacts of chronic paternal alcohol exposures revealed that male alcohol use is associated with dose-related changes in offspring stress-related behaviors (Abel, 1991). Similarly, recent studies by our group using a mouse model demonstrate dose-dependent effects on offspring placental weights and bodyweight normalized brain weights (Thomas et al., 2022; Thomas et al., 2023). However, no studies have determined if paternal alcohol use induces dose-dependent effects on offspring craniofacial shape and symmetry.

Geometric morphometrics is a landmark-based technique used to compare the proportional size, rotation, and relative positions of facial landmarks and to identify differences in shape and morphology between populations (Klingenberg, 2011; Zelditch et al., 2012; Katsube et al., 2022). In this procedure, generalized Procrustes analysis (GPA) removes scale from the dataset and standardizes all specimens by placing them into a common coordinate system. Subsequently, canonical variant (CV) analysis identifies the proportional relationships that best distinguish shape differences among groups (Klingenberg, 2011; Zelditch et al., 2012; Attanasio et al., 2013; Wiseman et al., 2021; Katsube et al., 2022; Sam Penrice, 2022). Accordingly, geometric morphometric analysis is widely employed to study diverse aspects of craniofacial patterning, including characterizing fetal alcohol syndrome-associated craniofacial dysmorphology in preclinical and clinical studies (Kaminen-Ahola et al., 2010; Klingenberg et al., 2010; Mutsvangwa et al., 2010). Here, we employed geometric morphometric analyses to determine dose-dependent changes in facial shape in the gestational day 16.5 male offspring of alcohol-exposed sires.

## Methods

### Animal husbandry and alcohol treatments

Our previous publication (Thomas et al., 2022) provides detailed descriptions of the preconception alcohol treatments, male breeding, and tissue collections. Briefly, we used C57BL/6J mice (Strain #:000664 RRID: IMSR_JAX:000664), which we housed in the Texas A&M Institute for Genomic Medicine under a reverse 12-h light/dark cycle, with lights out at 8:30 a.m. and lights on at 8:30 p.m. We maintained mice on a standard diet (catalog# 2019, Teklad Diets, Madison, WI, United States), adding additional cage enrichments to minimize stress, including shelter tubes for males and igloos for females (catalog# K3322 and catalog# K3570, Bio-Serv, Flemington, NJ, United States).

We acclimated male mice to individual housing conditions for 1 week, then employed a prolonged version of the Drinking in the Dark model of voluntary alcohol consumption. Beginning 3 h into the dark phase, we exposed postnatal day 90 male mice to one of four preconception treatments by replacing the water bottle of their home cage with a bottle containing either 0% (Control), 3% (Low-dose), 6% (Medium-dose), or 10% (High-dose) w/v ethanol (catalog# E7023; Millipore-Sigma, St. Louis, MO, United States). We simultaneously exchanged the water bottles of Control and ethanol-exposed males to ensure identical handling and stressors. We exposed males to these treatments for 4 h every day, maintained the treatments for 6 weeks, then began mating exposed males to naive dams but maintained the preconception treatments during this period.

We recorded treatment bottle weights and sire bodyweights weekly. We then quantified weekly fluid consumption by calculating the grams of fluid consumed divided by sire body weight (g/g). Subsequently, we determined the daily ethanol dose (g/kg) by multiplying the weekly fluid consumption (g/g) by the treatment group dose (0.03, 0.06, or 0.10) and then divided this number by 7 days. For consistency with clinical studies (Leeman et al., 2010), we converted this number to grams per kilogram (g/kg).

We bred exposed males to naive postnatal day 90 C57BL/6J females by synchronizing the female reproductive cycle using the Whitten method (Whitten et al., 1968), then placing the female in the male’s home cage immediately after the male’s daily exposure window. 6 h later, we confirmed matings by the presence of a vaginal plug and returned females to their original cages. We rested treated males for 72 h, during which the males continued their exposures and then used them again in a subsequent mating.

### Digital image acquisition and processing

We terminated pregnant dams on gestational day 16.5 using carbon dioxide asphyxiation followed by cervical dislocation. Subsequently, we dissected the female reproductive tract and collected digital photographs of each fetus within the litter. In curating this dataset, we included the litter I.D., sex, and uterine position of each fetus and then used the publicly available program **tpsUtil32** [(Rohlf, 2015); version 1.83] to generate a T.P.S. database. We then imported digital images of the front profile into the publicly available image analysis software **tpsDig2w64** [(Rohlf, 2005) version 2.32]. We set the reference scale bar in the picture to 1 mm, then demarcated the eighteen facial landmarks described by Anthony et al. (2010). To ensure consistency, a single individual (M.N.G) demarcated the landmarks in each photograph, consistently identifying the exact location and order for each image. We then created a linear outline around the head and digitized the landmarks and outlines as a T.P.S. file. **tpsDig2w64** (Rohlf, 2005) then adds additional landmarks, including the midpoints between features and other aspects of the outline, producing a total of 47 landmarks. As we did not anticipate that the Low-exposure treatment would impact craniofacial development, we collected additional Control and Low samples in a separate experimental cohort to ensure the reproducibility and robustness of our data (von Kortzfleisch et al., 2020; McGill and Threadgill, 2023).

### Geometric morphometrics and statistical analyses

We imported the generated T.P.S. files for each fetus into the MORPHOJ software [(Klingenberg, 2011) version build 1.07a, Java version 1.8.0_291 (Oracle Corporation)] and conducted geometric morphometric analysis. We added classifiers describing each treatment group and then separately normalized the datasets for scale, rotation, and translation using the Procrustes fit feature (Klingenberg, 2011). We then generated a covariance matrix, which we used to conduct Principal Component Analysis (PCA). Our PCA analysis revealed that PC1 and PC2 described most (∼50%) of the variation in our model.

We then used canonical variate (CV) analysis to identify differences in facial features between treatments and exported the raw CV scores into the Paleontological Statistics Software Package for Education and Data Analysis (PAST) analysis software (Hammer et al., 2022) version 4.03 [https://softfamous.com/postdownload-file/past/18233/13091/]. We conducted multivariate analyses of the raw CV scores using statistical methods described previously (Zelditch et al., 2012; Attanasio et al., 2013; Wiseman et al., 2021; Sam Penrice, 2022). These included the parametric Multivariate analysis of variance (MANOVA), and non-parametric Analysis of similarities (ANOSIM), and Permutational multivariate analysis of variance (PERMANOVA) tests, followed by Bonferroni correction. We generated the CV lollipop and wireframe diagrams, as well as scatter plots, using the graphing features of MORPHOJ (Klingenberg, 2011).

We calculated the average daily dose (Thomas et al., 2022) for the males that sired the offspring we examined in this study, then transferred these data into the statistical analysis program GraphPad Prism 9 (RRID: SCR_002798; GraphPad Software, Inc., La Jolla, CA, United States). We set the statistical significance at alpha = 0.05, used the ROUT test (Q = 1%) to identify outliers, and then verified the normality of the datasets using the Shapiro–Wilk test. We then used a one-way ANOVA followed by Tukey’s *post hoc* testing to contrast differences between treatments.

### Front landmark key

1. Top of head (Central, highest point of head, in line with the nose)
2. Bottom of mandible (Bottom-most point of jaw)
3. Right corner of mouth (Furthest right point where mouth closes)
4. Left corner of mouth (Furthest left point where mouth closes)
5. Top of philtrum (Closest point below the base of nose)
6. Bottom of philtrum (Ventral extent of philtrum, closest junction between two lips)
7. Tip of nose (top, central most part of the nose)
8. Nasion (Central point between the two eyes)
9. 3 O’clock position of left eye (medial-most part of the external eye)
10. 12 O’clock position of left eye (topmost part of the external eye)
11. 9 O’clock position of left eye (medial-most part of the external eye)
12. 6 O’clock position of left eye (bottom most part of external eye)
13. Pupil of left eye (Center of the left eye, lighter color)
14. 3 O’clock position of right eye (medial-most part of the external eye)
15. 12 O’clock position of right eye (topmost part of the external eye)
16. 9 O’clock position of right eye (medial-most part of the external eye)
17. 6 O’clock position of right eye (bottom most part of external eye)
18. Pupil of right eye (Center of the right eye, lighter color)

## Results

**FIGURE 1:**
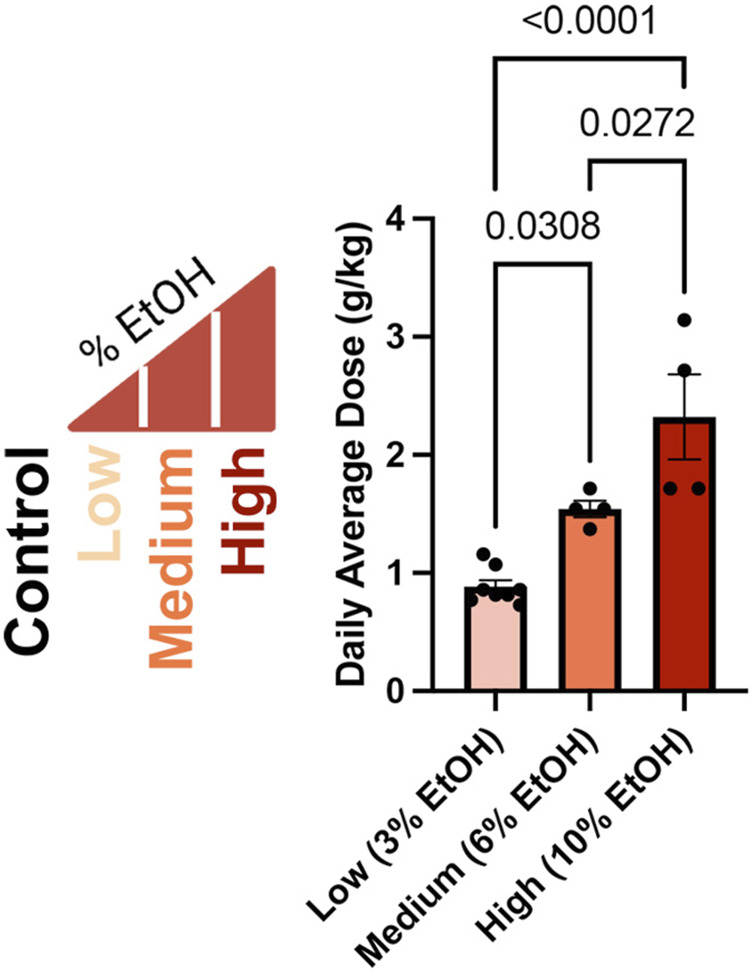
Average daily ethanol dose for males in each treatment group. We exposed males to different ethanol treatments for 6 weeks (Thomas et al., 2022), then determined the average daily dose of the males that sired the offspring we examined in this study as grams per kilogram per day (g/kg/day), consistent with clinical studies (Leeman et al., 2010). We used a one-way ANOVA to contrast differences between treatments; error bars represent the standard area of the mean, n = Low 8, Medium 4, and High 4.

In our previous studies examining the dose-dependent effects of preconception paternal alcohol exposures on offspring fetoplacental growth, we exposed Control males to water alone and administered Low (3%), Medium (6%), and High (10%) ethanol (w/v EtOH) treatments using the voluntary Drinking in the Dark exposure model (Figure 1) (Thomas et al., 2022). In these previous studies, we strictly enforced consumption rates and eliminated males that dropped below a weekly fluid consumption of 0.08 g/g/week, one standard deviation below a previously identified average (Chang et al., 2019). We eliminated one Medium-dose and ten High-dose (10%) males from this original cohort. For the males that sired the offspring we examined in this study, the average daily dose for each treatment was 0.8839 (Low), 1.543 (Medium), and 2.321 (High) g/kg/day. We observed significant increases in the average daily ethanol dose across each of the Low, Medium, and High treatments (Figure 1).

**FIGURE 2:**
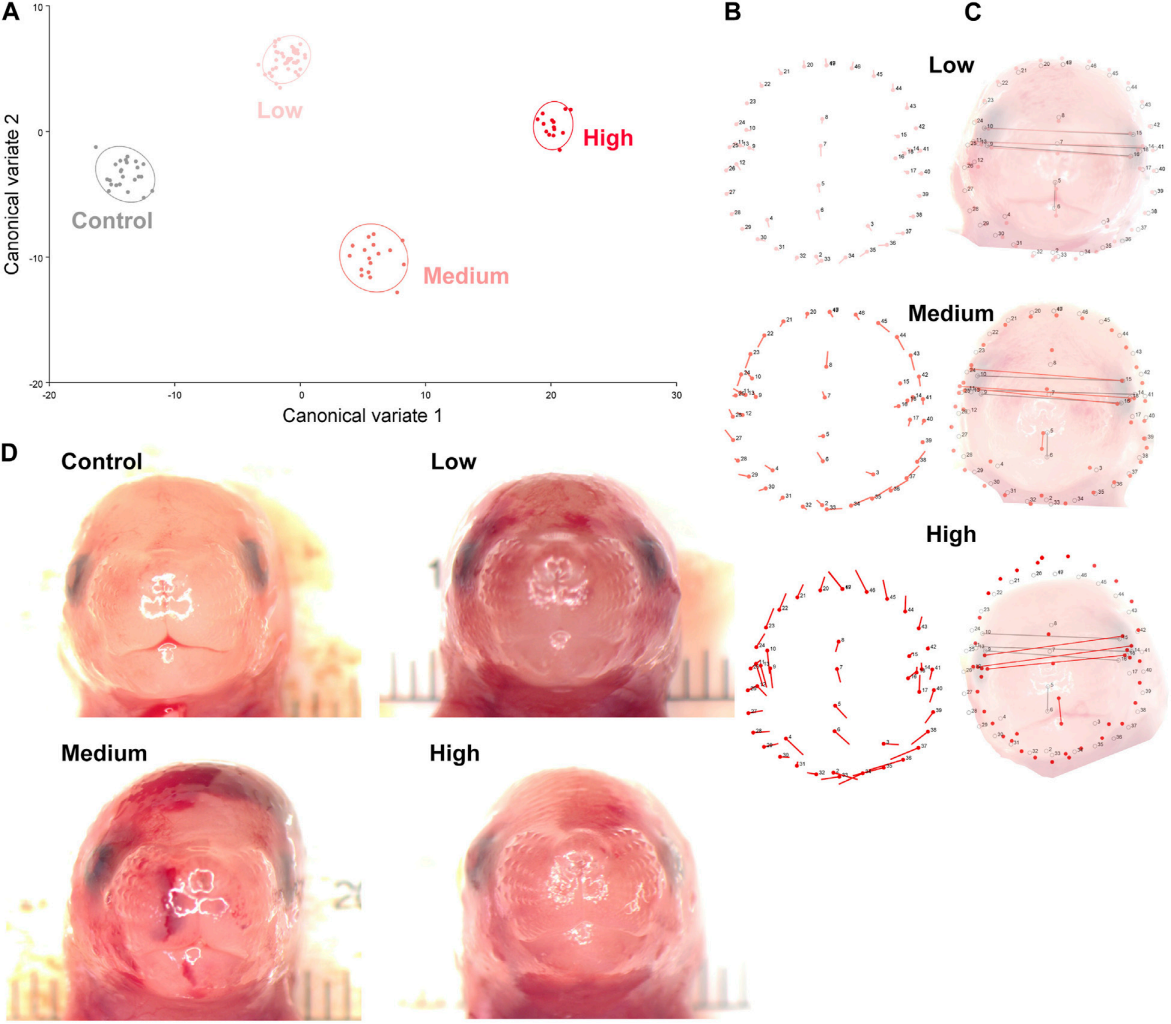
Preconception paternal alcohol exposures induce dose-dependent changes in offspring craniofacial shape and symmetry. **(A)** We used geometric morphometrics, followed by canonical variate analysis to compare alcohol-induced shifts in the facial shape of the gestational day 16.5 male offspring sired by Low- (3%), Medium- (6%), and High-dose (10%) males. Canonical variant one accounted for 67.3%, and variant two accounted for 20.9% of the observed variance. **(B)** Lollipop diagrams reporting relative shifts in facial landmarks (compared to Controls) induced by preconception paternal alcohol exposures between offspring derived from Low- (top), Medium- (middle), and High-dose (bottom) sires. **(C)** Wire diagrams comparing the shift in facial landmarks between the offspring of Low- (top), Medium- (middle), and High-dose (bottom) males. The red lines demarcate the alcohol-induced change, while the grey lines demarcate the average of the Control population. Note that representative pictures of the fetal face do not perfectly align with the population average, particularly in the High-dose treatments, where we observed wide variation in facial shape. **(D)** A selection of craniofacial images shows the observed dysmorphology range across treatment groups. We used digital images from 24 Control, 43 Low-, 17 Medium-, and 14 High-dose male fetuses, sampled from at least four litters.

Using digital images collected during this previous study, we used geometric morphometrics to compare the geometric relationships between eighteen craniofacial landmarks of gestational day 16.5 male fetuses sired by Control, Low-, Medium-, and High-dose males. We focused on the male fetuses as our previous studies revealed that male offspring exhibit a greater incidence of craniofacial dysgenesis than female offspring (Thomas et al., 2023). Procrustes ANOVA quantifies the relative amount of variation in size or shape attributable to one or more factors in a linear model. When comparing all treatments to each other, this analysis revealed that chronic preconception male alcohol exposure induced changes in shape (*p* < 0.0001) and centroid size (*p* = 0.0052), demonstrating a shift in facial allometry and center between treatments (Table 1). Canonical variant (CV) analysis of geometric facial relationships revealed that the Low-, Medium-, and High-dose treatments each induced distinct signatures of morphometric change, with rising dose increasing the shift along canonical variant one and canonical variant two, which together accounted for ∼88% of the variance we observed in our model (Figure 2A). We then used the raw CV scores to conduct three independent multivariate analyses, including MANOVA, ANOSIM, and PERMANOVA, followed by Bonferroni correction to identify significant differences in clustering and distance between treatment groups. Consistent with our CV plot (Figure 2A), each of these statistical tests identified significant differences between each treatment (*p* < 0.0006; Table 2; Supplementary Table S1).

**TABLE 1:** Procrustes Analysis of variance (ANOVA) comparing the impacts of paternal ethanol treatment on offspring shape and symmetry.

Comparison	Procrustes ANOVA (shape)	Centroid size (center)
All treatments	*p* < 0.0001	*p* = 0.0052
Control vs. Low	*p* < 0.0001	*p* = 0.6489
Control vs. Medium	*p* < 0.0001	*p* = 0.7482
Control vs. High	*p* < 0.0001	*p* = 0.0005
Low vs. Medium	*p* = 0.0825	*p* = 0.9918
Low vs. High	*p* = 0.0018	*p* = 0.0035
Medium vs. High	*p* = 0.0001	*p* = 0.0262

**TABLE 2:** Multivariate analysis of variance (MANOVA) of the raw canonical variant scores from each treatment.

MANOVA pairwise comparisons	Control	Low (3%)	Medium (6%)	High (10%)
Control		*p* = 2.1203E-54	*p* = 1.2945E-35	*p* = 1.1495E-37
Low (3%)	*p* = 2.1203E-54		*p* = 6.1671E-47	*p* = 5.3607E-50
Medium (6%)	*p* = 1.2945E-35	*p* = 6.1671E-47		*p* = 1.3949E-24
High (10%)	*p* = 1.1495E-37	*p* = 5.3607E-50	*p* = 1.3949E-24	

To better understand the dose-dependent effects of chronic paternal alcohol use on offspring facial shape and symmetry, we first used Procrustes ANOVA to conduct pairwise comparisons, contrasting the offspring of Control males with each of the paternal alcohol treatments. These analyses revealed that the Low and Medium preconception treatments each induced changes in overall shape (*p* < 0.0001). As in clinical studies (Klingenberg et al., 2010), CV analysis identified a shift of midline features to the right in offspring from the Medium treatment (Figures 2B, C). Notably, principal component analysis identified a right shift in the Low treatment but not Medium. However, compared to Controls, we did not observe any significant differences in centroid size for either treatment (Low *p* = 0.6489 and Medium *p* = 0.7482), indicating these changes did not shift the facial center (Figures 2B, C).

In contrast, using Procrustes ANOVA to compare male offspring derived from the Control and High treatment groups, we identified significant changes in both overall shape (*p* < 0.0001) and centroid size (*p* = 0.0005), indicating significant changes in facial allometry and a shift in the facial center. Consistent with our previous studies, these changes predominantly mapped to regions around the lower portion of the face, including the mandible (lower jaw) and maxilla (upper jaw) (Figures 2B–D). However, unlike our previous studies, changes induced by the High preconception treatment shifted midline features to the left and lowered the positioning of the right eye. Finally, we used Procrustes ANOVA to conduct pairwise comparisons between the Low and Medium and Low and High doses. These analyses did not identify differences in overall shape or centroid size between the Low and Medium treatments. In contrast, comparisons to the High treatment revealed significant changes in both overall shape (Low vs. High *p* = 0.0018; Medium vs High *p* < 0.0001) and centroid size (Low vs. High *p* = 0.0035; Medium vs. High *p* = 0.0262 (Table 1).

## Discussion

Clinicians and researchers consistently struggle to understand the enormous variation in FASD severity and cannot fully explain why some alcohol-exposed babies are affected while others are not. Even among co-exposed dizygotic twins, there is wide variation in the presentation of FASDs (Streissguth and Dehaene, 1993; Riikonen, 1994; Astley Hemingway et al., 2018). Therefore, although maternal alcohol use undoubtedly plays a causal role in developing FASDs, additional factors beyond maternal alcohol use must contribute to the etiology of this disorder. As emerging research demonstrates that paternal drinking is a plausible driver of alcohol-related phenotypes (Terracina et al., 2022; Rice et al., 2024), researchers must now rigorously examine paternal epigenetic contributions to this condition.

Using a mouse model, we sought to determine if, similar to mouse models examining maternal exposures (Petrelli et al., 2018), preconception paternal alcohol use induces dose-dependent changes in craniofacial development. Here, we employed geometric morphometrics to determine the ability of increasing paternal ethanol dose to induce changes in craniofacial symmetry and shape of the F1 male offspring. Our studies identified the intergenerational inheritance of alcohol-induced changes in facial shape, with progressive changes in allometry and a shift in facial symmetry and center corresponding with increasing dose. In support of this assertion, Procrustes ANOVA and canonical variant analysis identified a clear separation between treatments. Pairwise comparisons reveal a dose threshold between 1.543 and 2.321 g/kg/day. Below this threshold, preconception paternal alcohol exposure induces changes in facial shape, while above this threshold, paternal exposures alter both shape and centroid size. Notably, this range is consistent with, if not slightly lower than, reported doses inducing craniofacial dysgenesis in mouse models examining maternal alcohol exposures, which employed either constant 10% exposures or two acute IP EtOH doses of 2.9 g/kg [reviewed (Petrelli et al., 2018)].

Centroid size is the square root of the sum of squared distances of all landmarks from their center of gravity (obtained by averaging the x and y coordinates of all landmarks) (Klingenberg, 2016). Changes in this measure indicate a shift in the facial center and a loss of facial symmetry. As with clinical studies (Suttie et al., 2013; Abell et al., 2016; May et al., 2017; Blanck-Lubarsch et al., 2019; Blanck-Lubarsch et al., 2023), the alcohol-induced changes in facial shape we identified here predominantly centered on the lower aspects of the face, including the mandible and maxilla. Also, consistent with reports from the Parnell group using C57BL/6J mice (Parnell et al., 2014), we identified a right shift in many facial features and predominant impacts on the right eye. However, in offspring derived from the High treatment group, we identified a left shift in many features (see the lollipop diagrams in Figure 2B), suggesting differing paternal doses exert complex effects on offspring craniofacial development.

Although widely employed in examining gestational stressors, few studies have examined dose-response relationships between paternal exposures and adverse changes in offspring phenotypes. Previous studies examining paternal exposures to low- and high-dose mixtures of plastic-derived endocrine disruptors reported dose-related intergenerational and transgenerational impacts on offspring disease. However, the authors observed wide variation between sexes and different organ systems (Manikkam et al., 2013). As discussed above, we and Professor Abel observe dose-dependent impacts of alcohol on offspring fetoplacental growth and stress-related behaviors. These observations indicate that epigenetic changes induced by paternal exposures are unlikely binary (yes/no) but instead represent graded changes in epigenetic signals transmitting through sperm. Graded shifts in epigenetic transmission may help explain the wide variation in DNA methylation, patterns of histone retention/modification, and sperm-derived noncoding RNAs observed between studies examining paternal toxicants [reviewed (Van Cauwenbergh et al., 2020)].

Although we do not examine any potential epigenetic mechanisms of inheritance in this study, previous work in worms suggests that heritable changes in mitochondrial function drive graded alterations in offspring phenotypes (Durieux et al., 2011; Zhang et al., 2021). Our previous work demonstrates that paternal alcohol exposure causes lasting deficits in offspring mitochondrial function (Thomas et al., 2022). Mitochondria are critical regulators of cell fate and are particularly relevant to controlling neural crest cell (NCC) differentiation, which catalyzes the formation of the developing face (LaMantia, 2020). NCCs are extremely sensitive to mitochondrial dysfunction and the resulting redox imbalance (Fitriasari and Trainor, 2021). For example, redox imbalance potently disrupts Wnt signaling, a critical regulator of neural crest cell differentiation (Costa et al., 2021). Further, mitochondrial fusion is required for cells to undergo the epithelial-to-mesenchymal transition (Wu et al., 2019), and disruption of bioenergetic pathways in neural crest cells leads to growth arrest of the facial primordia, inducing midline orofacial defects (Nie et al., 2018), similar to those we observed in our model. We postulate that sperm-inherited epigenetic factors alter the embryonic control of mitochondrial and oxidative stress-related genetic pathways, disrupting neural crest cell function. Enrichment of these same mitochondrial and redox-related pathways in the reproductive tract and sperm noncoding RNA profile of alcohol-exposed males support this assertion (Roach et al., 2023).

Image analysis-based diagnostics are an emerging area of interest in our efforts to diagnose multiple developmental disorders, particularly those with subtle dysmorphia, including FASDs (Roomaney et al., 2022). Morphometric analysis is a powerful tool in these studies, particularly when combined with machine learning strategies (Blanck-Lubarsch et al., 2023). Although we recognize that paternal information is often missing or challenging to obtain, our studies suggest that incorporating paternal exposure history is essential to fully understand the developmental origins of FASD-related facial phenotypes. Further, our work and others demonstrate the need to expand public messaging to communicate the reproductive dangers of alcohol use to include both parents.

## Data Availability

The raw data supporting the conclusions of this article will be made available by the authors, without undue reservation.
