# Supplementary material for: Chronic paternal alcohol exposures induce dose-dependent changes in offspring craniofacial shape and symmetry
Source: Front Cell Dev Biol. 2024 Jul 1;12:1415653. doi: 10.3389/fcell.2024.1415653 (PMC11246915; doi:10.3389/fcell.2024.1415653)
Supplement: Supplementary file 1 [file Table1.DOCX]

**Supplemental Table 1. ANOISM and PERMANOVA of the raw canonical variant scores of each treatment.** Output generated using the Paleontological Statistics Software Package for Education and Data Analysis (PAST) software ((32) version 4.03; [https://softfamous.com/postdownload-file/past/18233/13091/.]).

**One-Way ANOISM**

| **Summary** |  |
| --- | --- |
| Permutation N | 9999 |
| Mean Rank Within | 703.5 |
| Mean Rank Between | 3080 |
| R | 1 |
| p-value | 0.0001 |

| **Pairwise (Bonferroni-corrected)** | | | | |
| --- | --- | --- | --- | --- |
|  | CM | LM | MM | HM |
| CM |  | 0.0006 | 0.0006 | 0.0006 |
| LM | 0.0006 |  | 0.0006 | 0.0006 |
| MM | 0.0006 | 0.0006 |  | 0.0006 |
| HM | 0.0006 | 0.0006 | 0.0006 |  |

**One-Way PERMANOVA**

| **Summary** |  |
| --- | --- |
| Permutation N | 9999 |
| Total Sum of Squares | 1.682E04 |
| Within-group sum of squares | 470 |
| F | 1090 |
| p-value | 0.0001 |

| **Pairwise (Bonferroni-corrected)** | | | | |
| --- | --- | --- | --- | --- |
|  | CM | LM | MM | HM |
| CM |  | 0.0006 | 0.0006 | 0.0006 |
| LM | 0.0006 |  | 0.0006 | 0.0006 |
| MM | 0.0006 | 0.0006 |  | 0.0006 |
| HM | 0.0006 | 0.0006 | 0.0006 |  |
